# Sometimes it’s good to be lucky: blood flow, glutathione, oxidative stress, and mitochondria

**DOI:** 10.3389/fphys.2025.1766585

**Published:** 2026-02-09

**Authors:** Walter Gay Bottje

**Affiliations:** Department of Poultry Science, Center of Excellence for Poultry Science, Division of Agriculture, University of Arkansas, Fayetteville, AR, United States

**Keywords:** mitochondria, blood flow, glutathione, oxidative stress, physiology

## 1 Luck begins

Much of the success I have had can be attributed to a good share of luck. I came out of high school interest in science – I’m not sure what I liked about it but I just wanted to learn more. I went to Eastern Illinois University (EIU) in a Pre-Professional route majoring in Zoology. I chose EIU because I wanted to swim competitively and not for any academic reason. I knew the Ray Padovan who had coached me in age-group swimming one summer. I lucked out because EIU had a strong biology program that helped me in graduate school and beyond. After my first semester, Padovan told me I would receive a tuition scholarship through the Athletic Department based on my GPA (not for my phenomenal swimming ability). I came out of Eastern with a BS in Zoology that included 5 physiology courses and a minor in Chemistry. Physiology was really what floated my boat but it took me awhile to get back to it. During my MS in ruminant nutrition, at Southern Illinois University (Carbondale, IL) because of a snowstorm, Amtrak Railroad and Interstate 57 were closed. If the snowstorm had not shut everything down, I would never have met my wife, Sari. This was a huge stroke of luck!

After the MS, I went to the University of Illinois for a PhD. After going through two labs (ruminant nutrition and rumen microbiology), I finally got back to my real interest in physiology with Dr. Paul Harrison (PCH). I would not recommend this path for graduate students as it added a couple years to my PhD program. I worked on heat stress physiology in broilers and ended up with 5 publications (Bottje W. G. and Harrison P. C., 1985; Bottje W. G. and Harrison P. C., 1985; Bottje W. G. and Harrison P. C., 1986; Bottje W. G. and Harrison P. C., 1986; Bottje and Harrison, 1987). However, the nearly 2-year delay was lucky because I ended up doing a post-doc that ultimately led to an NIH grant a couple years later. I interviewed at the University of New Hampshire but (luckily) I was not hired for that position. One thing that I lacked was post-doc experience.

When I put my PhD committee together, the ‘normal’ number of committee members was 5. I added a sixth member, Dr. Ken Holmes, in the Dept. of Veterinary Biosciences (U of I). Dr. Holmes had developed a new method of measuring blood flow in tissue called a thermal pulse decay (TPD) system. I was lucky enough to have been hired into his lab as a post-doc to work on further development and validation of the TPD system (see Arkin et al., 1986). The TPD method could measure time course changes in blood flow (tissue perfusion) at 3 min intervals in 6 areas in a single organ or multiple organs over several hours. Placement of probes in the renal cortex and medulla, enabled us to take repeated measurements in these regions of the kidney – something that had never been done before.

## 2 Contributions to science

### 2.1 Getting started

While working in Dr. Holmes’ lab, Dr. Hassan (in an adjacent lab), came in 1 day and mentioned that while working with a chemical that rapidly depletes hepatic glutathione (GSH)^1^ levels, he noticed that the rat’s ears turned red. He said, “*I think there are changes in blood flow happening – you should see if it affects liver blood flow”*. We conducted a set of studies on rats that revealed an inverse relationship between hepatic blood flow and tissue GSH. This study eventually led to an NIH grant.

I took a chance and gave my interview seminar for the Dept. Animal Science (University of Arkansas, UA) on the GSH and liver blood flow study in rats (even though the position was for an Environmental Physiologist in poultry). I was hired and started at the UA, Division of Agriculture in July of 1985. That fall, I attended the American Physiology Society (APS) fall meeting (Niagra Falls, CN) and presented a poster on the apparent inverse relationship between GSH and liver blood flow. Two people stopped by my poster that had a huge impact on me. The first one was Dr. Aubrey Taylor who was President of APS who thought the research was interesting and novel and encouraged me to pursue this rigorously. I submitted the GSH-liver blood flow manuscript one more time and got it accepted in Biochemical Pharmacology (Bottje et al., 1986). The second person I met was Dr. Bob Wideman (at Pennsylvannia State University) who was interested in the TPD method and how it might be used for studying blood flow mechanisms in the avian kidney. We ended up doing a series of studies a few years later using his one lobe avian kidney model. That was a very good piece of luck for me.

In early 1986, I gave a seminar for the Chemistry Department at UA the liver blood flow and GSH interrelationship. After the seminar, Dr. Collis Geren (Dept. Chair and later the Dean of the Graduate School) offered to help me package an NIH grant which was extremely fortunate because I had not written a grant proposal before – another piece of luck. Dr. Geren was world renown for his fundamental work in spider venom toxicity. Dr. Geren was also one of those people who looks out for the general good of the community and never self-serving. In 1987, the grant was ranked in the top 5% and fully funded by NIEHS. Starting off as an Assistant Professor with a 5 years large federal grant was extremely lucky. I was able to hire a couple of post doc and graduate students without relying on departmental funds.

### 2.2 Antioxidants, oxidative stress, blood flow and prostaglandins

To our knowledge, the inverse relationship between GSH and hepatic blood *in vivo* (Bottje et al., 1986) was the first time this was reported. Subsequently, we later reported that indomethacin, an inhibitor of prostaglandin synthase, attenuated both the increase in celiac blood flow in broilers (Beers et al., 1990) and hepatic blood flow and elevations in 6-keto PGF_1a_ (prostacyclin) in rabbits (Bottje et al., 1991) and swine (Bottje et al., 1992). There were also inverse relationships between GSH levels and prostaglandin synthesis in renal medullary homogenates (Nejad and Bottje, 1992). The increased blood flow in tissues following toxic insult could contribute to tissue damage as well as help in tissue repair and recovery.

### 2.3 Interorgan circulation of glutathione

A fundamental study by Anderson et al. (1980) described interorgan circulation of GSH that entailed synthesis in the liver followed by export into the general circulation and taken up by extrahepatic tissues. In that study, blood samples were taken at a single time point; ∼20 from a systemic artery and hepatic portal vein (representing afferent sources of blood entering the liver) with only 4 samples obtained from the hepatic vein which is difficult to reach due to its location next to the diaphragm within the thoracic cavity. Birds, however, do not have a diaphragm, thus it was possible to obtain repeated blood samples from the hepatic vein. This was facilitated with a hooked needle that Bob Wideman used in his avian kidney studies. In the study by Wang et al. (1998), interorgan circulation of GSH was clearly confirmed and was documented in the avian liver for the first time. This technique also enabled assessment effects of a stress hormone (norepinephrine) that stimulated GSH release from the liver (Song et al., 2000) and hepatic extraction of circulating amino acids and impact of methionine infusion across the hepatic vasculature (Song et al., 2001).

### 2.4 Oxidative stress, mitochondria, and pulmonary hypertension syndrome (PHS)

At the fall physiology meeting in 1986, Bob Wideman mentioned a new problem in the poultry industry that he had seen at altitude and was now showing up at sea level; ascites (PHS). I visited his lab after the meeting and he showed me evidence of lung damage in day old chicks. The GSH-oxidative stress studies associated with the NIEHS studies led to an interest in determining if oxidative stress was associated with PHS and presented in report by Enkvetchakul et al. (1993) and later to a study investigating Vit E and PHS (Bottje et al., 1995). This research also sparked an interest in mitochondrial function and biochemistry as mitochondria are a major site of endogenous oxidative stress. Site-specific defects in the electron transport chain that would contribute to higher oxidative stress were identified in liver (Cawthon et al., 1999), lung (Iqbal et al., 2001), and heart (Tang et al., 2002) obtained from broilers with PHS.

### 2.5 Mitochondria and feed efficiency

Interest in mitochondria continued with studies that revealed evidence of a link between muscle mitochondrial function and feed efficiency (FE). In a series of studies, evidence of mitochondrial dysfunction and/or biochemistry, including site-specific defects in electron transport, in tissues obtained from broilers expressing a low FE phenotype were identified in muscle (Bottje et al., 2002; 2009; Iqbal et al., 2001), duodenum (Ojano-Dirain et al., 2004; Ojano-Dirain et al., 2007), liver (Iqbal et al., 2001), lymphocytes (Lassiter et al., 2006), and heart muscle (Tinsley et al., 2010). Differences in proton leak kinetics were also determined in muscle mitochondria between high and low FE groups (Bottje et al., 2009).

### 2.6 Global gene expression and feed efficiency

Because mitochondrial ROS initiate signal transduction, we conducted global gene and protein expression analysis to understand the gene and gene product landscape associated with feed efficiency (Kong et al., 2011; 2016; Bottje et al., 2012, Bottje et al., 2017a; Bottje et al., 2017b). Interesting aspects of the story were that mitochondria in high FE animals appeared to have enriched ribosomal machinery and protein translation compared to muscle from low FE phenotype (Bottje et al., 2017b). Based on the global expression studies, further insight into fundamental mechanisms revealed that high FE birds exhibited enrichment of intracellular degradation pathways of autophagy and proteosomes (Piekarski-Welsher et al., 2018). This suggests that high FE animals may either repair proteins quicker or turnover damaged proteins to a greater extent than low FE. The myostatin signaling pathway was shown to play a role in the phenotypic expression of FE also (Lassiter et al., 2018). Using an analytical method of regulatory impact factor (RIF) analysis (Hudson et al., 2009; 2012; Reverter et al., 2010) identified progesterone as having a major influence on the phenotypic expression of FE (Bottje et al., 2017a). This led to work that clearly identified the presence of several hormone receptors including progesterone on avian mitochondria (Lassiter et al., 2018).

### 2.7 Water efficiency

Since 2019, I have been lucky to serve as project director (PD) on a USDA NIFA Sustainable Agriculture Systems project^2^. The “catalysts” for this were Dr.’s Dridi (UA) and Lei (Cornell University) coerced me into being the PD. One sub-aim on this project was headed up by Dr. Sara Orlowski (UA) who successfully selected broilers for low water conversion ratio (LWCR, water efficient) and high water conversion ratio (HWCR, water inefficient) from a modern random bred (MRB) base broiler population^3^ (Hiltz et al., 2021). A series of studies have been conducted to assess the effect that divergent selection has had on gene and/or protein expression in the hypothalamus, kidney, intestines, immune systems, and meat quality (see Aloui et al., 2024; Lassiter et al., 2024; Lassiter et al., 2025; Orlowski et al., 2024; Santamaria et al., 2025). While selection for feed conversion ratio (FCR) has a positive impact on water, selection for WCR has resulted in even further improvements in water use efficiency. These studies hopefully will provide a way to help the poultry industry down the road as water scarcity becomes even more prevalent in the face of hotter temperatures over longer periods of time.

## 3 Summary

Have I been lucky? Absolutely! Many events have put me in the right place at the right time. I have been fortunate to have had great graduate students, post-docs and collaborators. I was part of the development of a Center that brought in some of the best faculty in poultry science. I also want to acknowledge contributions by Dr. Kentu Lassiter who has been working in the lab since he was an undergraduate student. Finally, I’ve also been extremely lucky to have received funding throughout my career through various Federal agencies, State, and industry sources.

## Footnotes

1 Glutathione is an endogenous antioxidant found at mM levels in most cells. It plays vital roles in numerous redox reactions in the cell.

2 USDA NIFA SAS 2019-69012-29905. Empowering the US Broiler Industry for Transformation and Sustainability.

3 The modern random bred (MRB) line was established in 2016 and represented a composite of commercial broiler lines at that time.
